# Preclinical evaluation of molecularly targeted fluorescent probes in perfused amputated human limbs

**DOI:** 10.1117/1.JBO.28.8.082802

**Published:** 2023-01-05

**Authors:** Logan M. Bateman, Kendra A. Hebert, Jenna A. Nunziata, Samuel S. Streeter, Connor W. Barth, Lei G. Wang, Summer L. Gibbs, Eric R. Henderson

**Affiliations:** a Dartmouth Health, Department of Orthopaedics, Lebanon, New Hampshire, United States; b Dartmouth College, Thayer School of Engineering, Hanover, New Hampshire, United States; c Dartmouth Health, Heart and Vascular Center, Lebanon, New Hampshire, United States; d Oregon Health and Science University, Department of Biomedical Engineering, Portland, Oregon, United States; e Dartmouth College, Geisel School of Medicine, Hanover, New Hampshire, United States

**Keywords:** fluorophore, fluorescence-guided surgery, lead agent selection, amputation

## Abstract

**Significance:**

This first-in-kind, perfused, and amputated human limb model allows for the collection of human data in preclinical selection of lead fluorescent agents. The model facilitates more accurate selection and testing of fluorophores with human-specific physiology, such as differential uptake and signal in fat between animal and human models with zero risk to human patients. Preclinical testing using this approach may also allow for the determination of tissue toxicity, clearance time of fluorophores, and the production of harmful metabolites.

**Aim:**

This study was conducted to determine the fluorescence intensity values and tissue specificity of a preclinical, nerve tissue targeted fluorophore, as well as the capacity of this first-in-kind model to be used for lead fluorescent agent selection in the future.

**Approach:**

Freshly amputated human limbs were perfused for 30 min prior to *in situ* and *ex vivo* imaging of nerves with both open-field and closed-field commercial fluorescence imaging systems.

**Results:**

*In situ*, open-field imaging demonstrated a signal-to-background ratio (SBR) of 4.7 when comparing the nerve with adjacent muscle tissue. Closed-field imaging demonstrated an SBR of 3.8 when the nerve was compared with adipose tissue and 4.8 when the nerve was compared with muscle.

**Conclusions:**

This model demonstrates an opportunity for preclinical testing, evaluation, and selection of fluorophores for use in clinical trials as well as an opportunity to study peripheral pathologies in a controlled environment.

## 1 Introduction

Fluorescence-guided surgery (FGS) is a nascent field aiming to improve the safety and efficacy of surgery through fluorescent labeling of important tissues using molecules called fluorophores. Because no therapeutic effect is desired, a fluorophore’s clinical utility is evaluated based only on its safety and ability to highlight the tissue of interest. Therefore, fluorophore dosing may be substantially lower than that for therapeutics, and toxicity is rarely a problem. The fluorophore binding specificity ratio and signal-to-background ratio (SBR) are key metrics when evaluating a potential lead agent. However, advancing to human trials through the Food and Drug Administration (FDA) phase 0/microdose pathway still requires substantial resources, including single-species pharmacokinetic and toxicity testing, which are significant financial investments.

There are currently four nontargeted, FDA-approved fluorophores for clinical use: indocyanine green (ICG), fluorescein, 5-aminolevulinic acid, and methylene blue (MB).^1^ Approved targeted probes include Cytalux and LUM015 (Lumicell, Newton, Massachusetts, United States). Although both ICG and MB have several indicated clinical uses,^2^^–^^12^ they are untargeted and conform to the lumen of the structures into which they are injected, usually blood vessels.^2^^–^^4^ As FGS has increased in popularity, there is growing demand for targeted fluorophores, which increase the contrast of specific tissues (e.g., Cytalux).^13^^,^^14^ The creation of fluorophore libraries through systematic modifications of a fluorophore scaffold is a method for improving desirable qualities, such as binding specificity, tissue uptake, and spectral matching to existing clinical imaging systems.^1^ Although many new near infrared agents demonstrate clinically advantageous performance in animal models, translation to the clinic requires rigorous safety testing and trialing.^15^ The high cost of successful translation—∼$19M for therapeutic trials^15^—creates an incentive to select lead agents with a strong likelihood for clinical success. We believe that understanding fluorophore performance in viable human tissue will enhance the accuracy and safety of lead agent selection, thereby reducing cost, time, and potential harm to patients.

Our group has partnered with the Gibbs Lab at Oregon Health and Science University. Our team has more than 10 years of experience designing and developing human nerve-specific fluorophores, resulting in a library of >200 candidate compounds to date. In preparation for lead agent selection, we developed a first-in-kind model for fluorophore testing in amputated human lower limbs to facilitate authentic evaluation. Herein we report on our initial experience and the technical details of this model in a single patient from our initial 10-patient proof-of-concept pilot study.

## 2 Methods

The Dartmouth Health Institutional Review Board approved this protocol after full review; informed consent was obtained from participants. Candidates for participation were identified through review of institutional surgical rosters. The inclusion criterion was patients receiving a lower-limb amputation for nononcological indications. Most patients’ amputations were indicated for vascular insufficiency or chronic infection, including nonhealing wounds associated with diabetes mellitus. The exclusion criteria included (1) patients undergoing limb amputation for oncologic indications and (2) patients with peripheral arterial disease that precluded arterial cannulation.

**Fig. 1:**
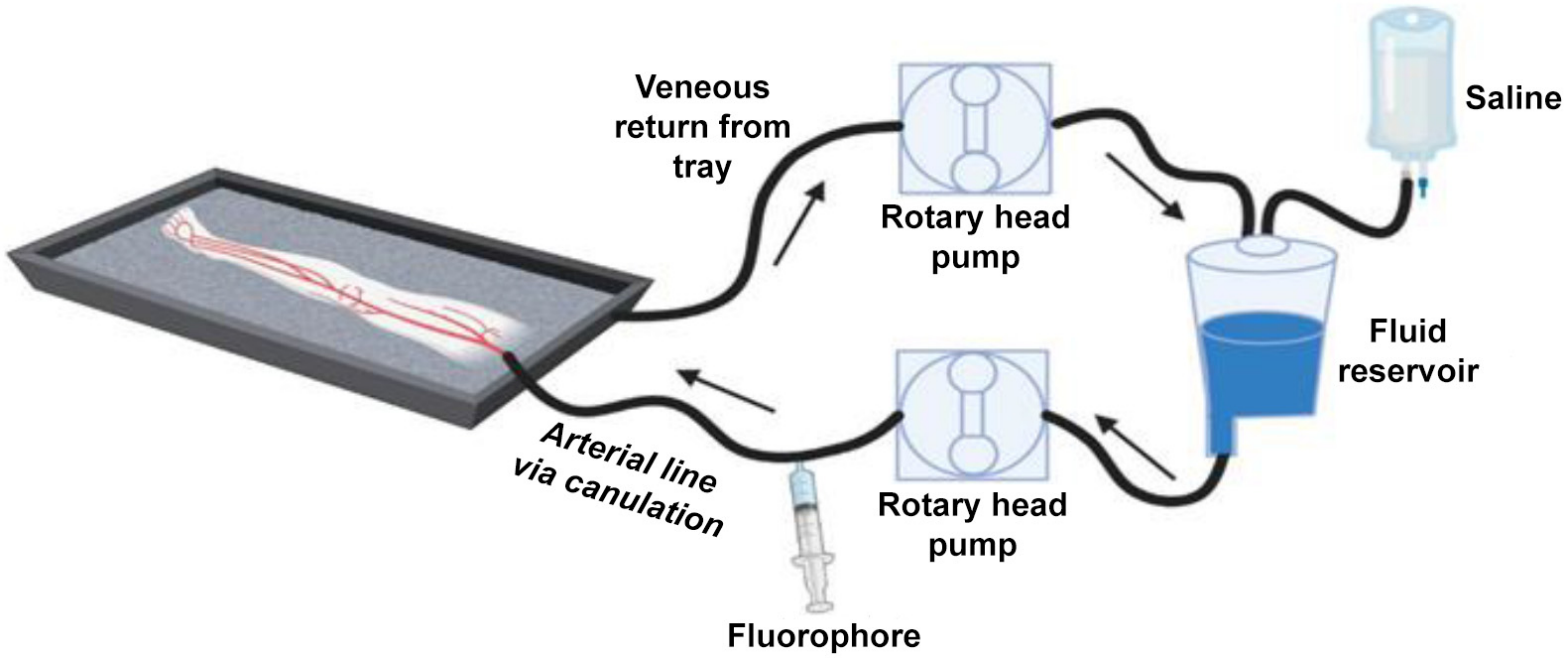
Schematic layout for perfusion of the amputated human limb model.^16^

Figure 1 illustrates the experimental setup used in this study. Following standard-of-care amputation, limbs were transported immediately to our surgical laboratory, where a dominant artery (superficial femoral, anterior tibial, or posterior tibial), based upon the level of amputation, vessel diameter, and vessel patency, was dissected and cannulated using a sized vascular cannula (Medtronic, Biomedicus, Minneapolis, Minnesota, United States). Each cannula was secured using a #0 silk suture and was connected to a perfusion circuit (Stockert, 3S, Breisgau, Germany). Limbs were then perfused with sterile saline at 90 to 170  mL/min to maintain physiologic pressures below 120 mm Hg. After confirming successful perfusion, a standard dose (1  mg/mL concentration and 10 mL total) of a nerve-specific fluorophore (LGW16-03) was administered to the limb through injection into the circuit via a Luer lock. Perfusion continued for 10 min, whereupon the perfusate was changed to sterile saline for model washout. Instead of venous cannulation for return, limbs were drained by gravity, and the perfusate was collected in a reservoir and then recycled into the circuit. Cardiac pump tubing (Sorin Group USA, SMART RX ¼” × 1/16,” Arvada, Colorado, United States) was used for the return and cannula lines, and an in-line pressure monitor was used to maintain appropriate physiological pressures. Following the conclusion of the perfusion period, the compartment supplied by the dominant cannulated artery was dissected, and the dominant nerve (common peroneal or tibial) was exposed for imaging; for this representative patient, the common peroneal nerve was imaged. *In situ* fluorescence imaging occurred via an open-field imager (Solaris, Perkin-Elmer, Waltham, Massachusetts, United States), which was set to excite and image at 660 nm. Samples of the target nerve and neighboring tissues (e.g., muscle, adipose, and fascia) were harvested for high-resolution fluorescence scanning using the Odyssey CLx (LI-COR Biosciences, Lincoln, Nebraska, United States), which was set to the 700-nm channel. Histopathology staining was performed on all nerve samples for examination of the tissue structure and nerve health. Following imaging and tissue sampling, the amputated limbs were taken to the Department of Pathology for standard clinical processing.

## 3 Results

**Fig. 2:**
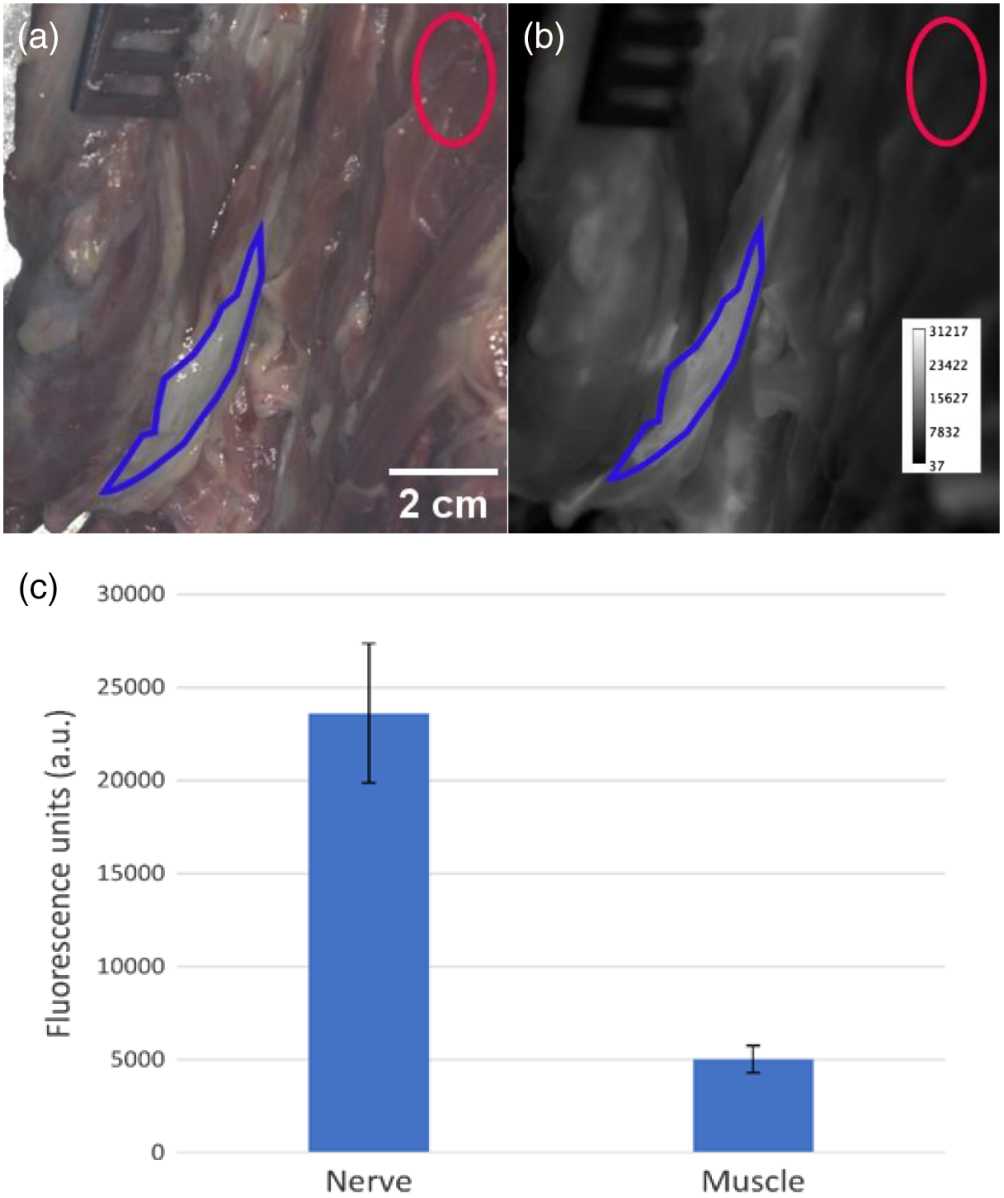
Common peroneal nerve outlined in blue (a) with white light and (b) fluorescence images following fluorophore administration. The red region of interest defines the signal of homogenous muscle tissue. (c) *In situ* imaging using an open-field imaging system shows a nerve-to-muscle ratio of 4.7.

**Fig. 3:**
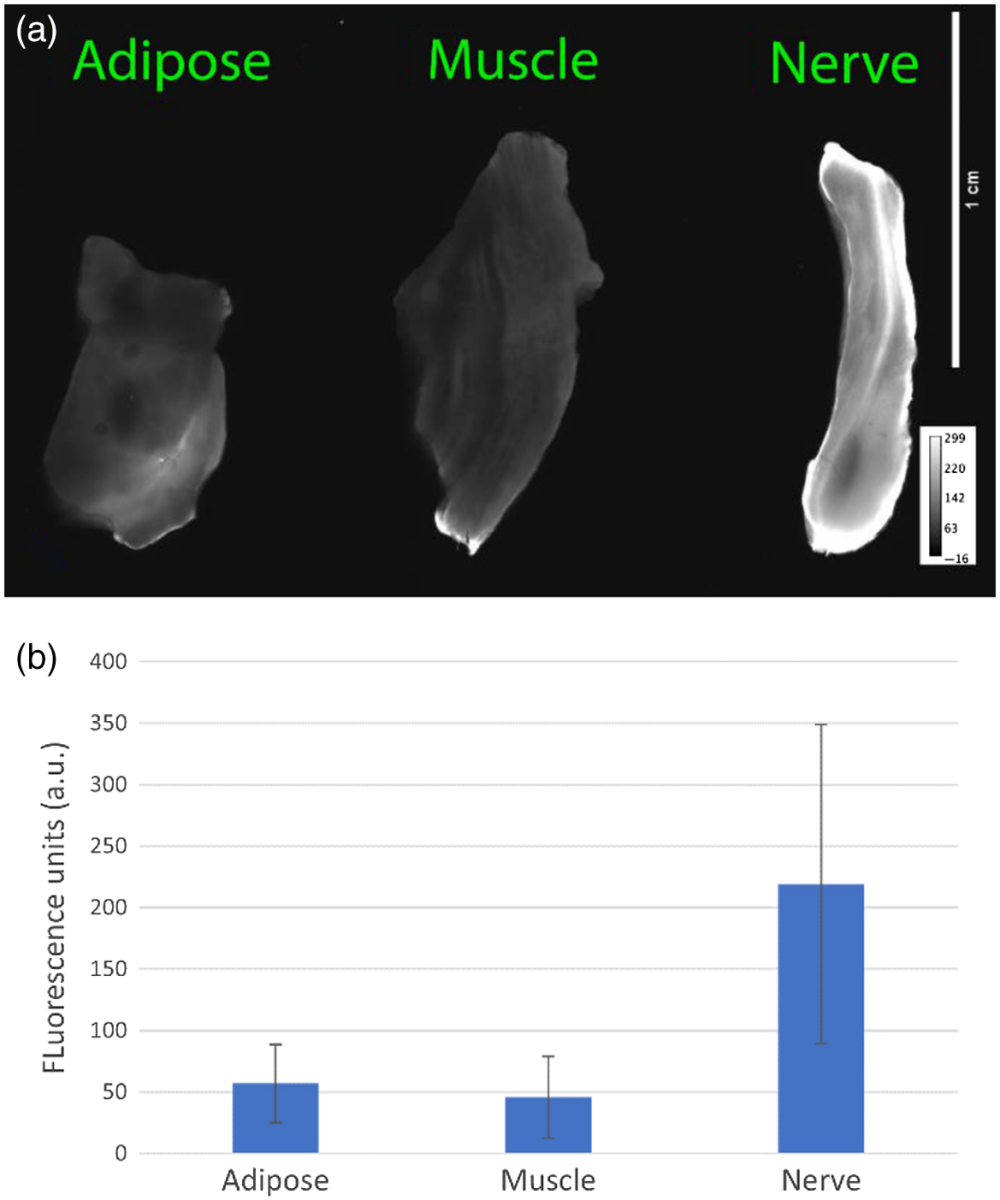
(a) From left to right, representative adipose, muscle, and peroneal nerve samples are pictured. Images were acquired with a closed-field fluorescence imaging system. (b) The SBR of the peroneal nerve sample compared with adipose was 3.8, whereas the SBR of the peroneal nerve sample compared with muscle was 4.8.

Results from a single, representative patient are reported herein. A 60-year-old man diagnosed with poorly controlled type II diabetes, occlusive peripheral artery disease, and recent burns to the lower extremity received a below-knee amputation due to the development of a soft-tissue infection. For this case, the peroneal artery was cannulated using a 10 French vascular cannula. Following cannulation, the limb was perfused with saline containing 10 mL of fluorophore for 10 min. This was immediately followed by a 20-min washout period using normal saline. After conclusion of the perfusion, the anterior compartment was dissected, and the deep peroneal nerve was exposed (Fig. 2). This was imaged using the open-field Solaris imaging system at an excitation wavelength of 660 nm. The regions of interest for wide-field imaging were chosen by identifying a nearby region of homogenous muscle tissue to compare against the nerve. The nerve was then sharply dissected using a surgical scalpel from the surrounding tissue along with adjacent muscle from the tibialis anterior as well as adipose tissue from between the muscular compartments. These resected samples were imaged on the closed-field Odyssey M imaging system in the 700-nm wavelength channel (Fig. 3).

## 4 Discussion

Using amputated human tissues for testing of drugs and as a model to understand disease is not entirely novel.^17^^,^^18^ Previous investigators described applications using skin cells grown or harvested from surgical patients. Our current model makes use of tissue in an acute setting prior to breakdown caused by ischemic muscle death, a process that takes three hours to begin and six hours to become irreversible.^19^ The use of normal saline in combination with a cardiac perfusion pump allowed us to mimic the osmotic and vascular pressure of an *in situ* physiologic system. The field of transplant medicine has included cadaveric work to enhance clinical strategies for organ procurement, sustenance, transport, implantation, and maintenance. A study published in the New England Journal of Medicine estimates that 80% of eligible lung transplant donors are rejected for transplant following organ procurement for a variety of indications.^20^ These organs often become eligible for research use, focused on improving outcomes of organ transplant patients, increasing perfusion time, or reducing rejection rates.^19^^–^^21^ Existing models of *ex vivo* limb perfusion exist,^22^^,^^23^ but these models were designed to study and improve limb transplantation, an altogether different indication. The objective of the present study is to perform whole-limb testing to evaluate preclinical fluorophore contrast, tissue toxicity, and tissue specificity as a surrogate for intravenous administration of novel probes to humans. This additional testing is important as protein expression varies across species and, therefore, may alter fluorophore performance when translated from animal models to human use.^24^^–^^28^

The limitations of this model include a lack of an oxygen carrier or nutrients to sustain the limbs for periods of time longer than 2 hrs. Without oxygenating and examining the limb, we cannot verify tissue viability or function and what effects this may have on our tissue-specific fluorescence intensity values. To address this limitation, we plan to adopt a perfusion protocol that will provide oxygen and nutrients to the limb while we perform testing in future studies.^22^^,^^29^^,^^30^ Sustaining a limb in this fashion will require not only feeding and oxygenating the limb appropriately but also maintaining the harmful metabolites and regular monitoring to ensure functional viability of the limb.^30^ Existing protocols used for upper extremity transplant surgery provide a useful basis for normothermic limb perfusion, but they are limited by short perfusion times and minimal use. The evolving field of vascular composite allografts (VCAs) has yet to be widely adopted,^31^ and no trials to date have exceeded 10 patients. These protocols also present a fairly substantial additional cost. Improvement of this model and advances in the field of VCA will increase perfusion times and may allow for not only further testing of fluorophores but also studying disease and acute pathologic changes caused by fracture or tumor presence.

## 5 Conclusion

The field of FGS in clinical medicine is growing, which has led to increased interest in novel fluorophore development. Due to the nontherapeutic nature of fluorophore use in clinic, lead agent selection is based primarily on optical performance to highlight target tissues. To improve lead agent selection, we have developed a first-in-kind, *ex vivo* model for testing fluorophore performance using amputated human limbs. This model may provide the ability for lead agents selected from initial rodent models to be evaluated in viable human tissues during the preclinical phase, demonstrating cross reactivity without risk of human harm. The improvement and physiological validation of this model could yield a valuable tool for expediting fluorophore development with lower costs and better ultimate drug performance. Although beyond the scope of this study, this model also represents a potential opportunity for exploration of disease epidemiology in human tissue and validation of FGS techniques in a simulated surgical environment.
